# A systematic review on indirect costs related to loss of productivity after stroke

**DOI:** 10.1186/s13561-026-00727-x

**Published:** 2026-01-31

**Authors:** Adrián Martín-Gutiérrez, Luis Leal-Vega, María Begoña Coco-Martín, Verónica Olmedo-Vega, Juan F. Arenillas-Lara

**Affiliations:** 1 Department of Medicine, Dermatology and Toxicology, Group of Applied Clinical Neurosciences, University of Valladolid, Av. Ramón y Cajal, 7, Valladolid, 47005 Spain; 2 Department of Sociology and Social Work, Faculty of Education and Social Work, University of Valladolid, Valladolid, Spain; 3 Department of Neurology, Stroke Unit & Stroke Program, Hospital Clínico Universitario de Valladolid, Valladolid, Spain; 4 Social Work Unit, Hospital Clínico Universitario de Valladolid, Valladolid, Spain

**Keywords:** Stroke, Economic burden, Indirect cost, Productivity loss

## Abstract

**Background and objective:**

Stroke is a leading cause of death and disability worldwide. The age of stroke cases is decreasing, resulting in a loss of productivity for patients and their informal caregivers. However, socioeconomic impact analyses of stroke often underestimate the importance of these indirect costs. The objective was to conduct a systematic review of indirect costs associated with lost productivity after stroke to highlight their relevance.

**Methods:**

A systematic review and meta-analysis were conducted according to PRISMA guidelines. PubMed, Scopus, and Web of Science were searched for scientific publications analyzing loss of work productivity during first post-stroke year. To perform the comparative analysis, the type of stroke was taken into account in relation to the type of productivity loss and the age of the patients. The risk of bias was assessed with Consolidated Health Economic Evaluation Reporting Standards (CHEERS).

**Results:**

A total of 928 records were identified, of which 24 were included in this review. Lost productivity of patient during the first year after stroke onset had a mean value of $6057.98, representing 35.1% of total cost during this period. Patient´s lost productivity was higher in hemorrhagic stroke compared to ischemic stroke ($12,783.17 vs. $8,239.34). In contrast, lost productivity of informal caregivers was slightly higher in ischemic stroke than in hemorrhagic stroke ($5,628.26 vs. $4,205.25). Patients aged 18–44 years had the highest work productivity loss ($16,114.33).

**Conclusions:**

Indirect costs represent a significant part of the economic burden of stroke and should therefore be included in future socioeconomic impact studies.

**Supplementary Information:**

The online version contains supplementary material available at 10.1186/s13561-026-00727-x.

## Introduction

Stroke is a disease of great social impact worldwide, as it is the second leading cause of death and the main cause of disability in adults. The World Health Organization (WHO) estimates that every year there are 15 million new cases of stroke, of which 5 million die and another 5 million suffer permanent disability [1]. Strokes are classified into two types: ischemic and hemorrhagic, with ischemic strokes accounting for about 85% of all cases [2]. By the year 2045, the number of new stroke cases worldwide is expected to increase by 13%, mainly due to population aging and associated risk factors [3].

In recent decades, significant improvements in stroke prevention and treatment have been achieved, which have contributed to a decrease in mortality [4]. Globally, about 80% of people who suffer a stroke survive and more than 110 million people live with sequelae and disability after stroke that cause an impact at personal, family and social levels [5]. Based on this, the increasing incidence of stroke worldwide will lead to a higher number of survivors with long-term loss of functional capacity [6].

The economic impact of stroke from a social perspective includes direct and indirect costs [7] and its overall assessment with all these costs is obtained through cost of illness analysis. Specifically, direct costs are related to the hospital resources used in the acute phase (health costs), as well as the resources associated with the consequences generated after hospital discharge (non-health or social costs) [8]. On the other hand, indirect costs are related to the loss of productivity due to unemployment or reduced working hours, the cost to informal caregivers and the cost of premature mortality [9]. In this regard, it has been estimated that stroke has a socioeconomic impact of €64 billion annually only in Europe [10], 21% of which is due to lost productivity costs [11].

Work productivity is a source of wealth for society that is severely affected by stroke [12], since, although stroke predominantly affects adults over 60 years of age, a quarter of cases affect working-age persons younger than 65 years of age. In fact, the incidence of stroke has increased in the last decade by almost 16% among people aged 45 to 64, and by almost 15% among people aged 18 to 44 [13]. Productivity loss is generated by the temporary or permanent interruption of the patient’s work activities [14]. Specifically, there are productivity losses associated with the reduction or loss of the individual’s work capacity and associated with loss of production due to death [15].

Strokes in working-age adults lead to absenteeism during hospital stay and subsequent sick leave [16] and, after returning to work, some patients require temporary or permanent reduction in working hours depending on their functional status, resulting in presenteeism [17]. In addition, some patients may be forced to leave work prematurely due to severe disability after stroke [18]. Likewise, the patient’s environment, understood as family and close friends, may also experience a loss of work productivity due to the time dedicated to unpaid informal care of the stroke patient [19].

Customised surveys for patients and their environment [20], following the human capital or frictional cost approach, are commonly used to assess the loss of work productivity [21]. Primarily, the human capital method considers the productivity cost as a function of the patient’s salary by analysing the difference between the value of production before and after the disease [22], while the frictional cost method assesses the cost of replacing the vacant position with a new employee [23]. In addition, stroke patients may receive monetary benefits from the public sector as compensation for the reduced or lost ability to work normally [24]. However, analyses of the socioeconomic impact of stroke often underestimate the importance of indirect costs related to reduced or lost work productivity of the individual and their informal caregivers, or do not include these costs [25]. In this regard, recent systematic reviews analysing costs during the long-term post-stroke phase have confirmed that indirect costs are often left out of the analyses [26].

A systematic review published more than a decade ago analysing studies that did include indirect costs of stroke between 1990 and 2012 concluded that these accounted for 33% of the total stroke cost, with a productivity loss of between $2,960 and $22,243 per patient per year [27]. In the absence of recent studies analysing the indirect costs of stroke, we considered it appropriate to conduct an updated systematic review on studies published since 2013 with the aim of highlighting the importance of the indirect costs of stroke and encourage their inclusion in future socioeconomic impact studies. In this sense, our systematic review included all studies that performed some type of analysis of indirect costs, either as part of the cost of illness analysis or as a specific analysis of indirect costs, in order to obtain a broader view of the results.

## Methods

This review followed the requirements of the Preferred Reporting Items for Systematic Reviews and Meta-Analyses (PRISMA) 2020 [28] and was registered in the PROSPERO International Prospective Register of Systematic Reviews (ID: CRD42025648286). No specific protocol was previously published for this systematic review. In addition, an analysis of heterogeneity was performed among using the random effects model with the inverse variance method, with the Tau² test, the Chi² test, and the I² statistic [29].

### Eligibility criteria

This systematic review included scientific publications that conducted an analysis to determine the indirect costs associated with loss of productivity after stroke. It should be noted that the term indirect cost encompass some type of work productivity loss due to absenteeism and/or presenteeism of the patient in their workplace, and/or time spent by informal caregivers caring for the patient instead of working. Thus, all studies that performed some type of analysis of indirect costs for at least one year after stroke were included, either as part of the total cost of illness analysis or as a specific analysis of indirect costs.

The search criteria included studies written in English published from 2013 onwards that dealt with stroke patients > 18 years, including any type of stroke, severity and location, and analyzed indirect costs for at least one year in the post-stroke phase. Regarding the design of the studies, there were no restrictions on sample size, retrospective or prospective type, perspective, or indirect cost analysis approach. However, studies that did not track indirect costs for at least one year after the stroke were excluded, as were those that assessed indirect costs in aggregate population terms and did not report the cost per individual patient.

### Sources of information

The electronic databases PubMed, Scopus and Web of Science (Wos), and clinical trial registries clinicaltrials.gov (https://clinicaltrials.gov/) and the International Clinical Trials Registry Platform (ICTRP) search portal were searched up to February 2025. These sources of information were used due to their rigor and broad coverage: PubMed contains more than 39 million biomedical references [30], Scopus indexes nearly 27,000 journals [31], WoS indexes more than 34,000 journals [32], ClinicalTrials.gov contain more than 500,000 registered studies [33], and the International Clinical Trials Registry Platform (ICTRP) integrates more than 600,000 registries [34]. These sources allowed for the retrieval of published evidence and ongoing trials, ensuring the comprehensiveness of the results of this systematic review.

In addition, to expand the identification of studies via other methods, a search was conducted for dissertations and theses in Proquest [35], grey literature in OpenGrey [36], and institutional and policy reports in PolicyCommons [37].

### Search strategy

Search terms were set according to the National Library of Medicine’s Medical Subject Headings (MeSH) terms 2025 and included: Stroke, Cerebrovascular Infarction, Indirect Costs, Indirect Cost Analysis, Indirect Cost Measurement and Indirect Cost Allocation. In this sense, the term “indirect costs” include the loss of work productivity due to post-stroke patient absenteeism and/or presenteeism, and/or the burden on informal caregivers related to the hours dedicated to patient care. Therefore, the articles included in this search refer to indirect costs under all these concepts. The search strategy in the electronic databases is presented in Supplementary file 1, and, for the search in the trial registers, advanced search sections were used. For the identification via other methods, an analysis of the bibliographic references of the included articles was carried out, as well as a search in other selected information sources.

### Selection process

The search of electronic databases and trial registries yielded a total of 928 results, which were then exported to EndNote 20 (Clarivate Analytics, Philadelphia, USA) for automatic removal of duplicates, leaving a total of 514 unique publications. Two reviewers (LLV and AMG) independently screened titles, keywords and abstracts for potential articles for inclusion. Disagreements were resolved by involving a third reviewer (MBCM) to reach consensus. Of 78 records sought for retrieval, 19 reports were not retrieved because the full text was not freely available, leaving 59 articles to be assessed for eligibility. After excluding non-English language articles and editorials, notes, letters, reviews and study protocols, a total of 8 trial records and 14 articles were included in the review, whose citation searching yielded two additional articles that meet eligibility criteria.

**Fig. 1:**
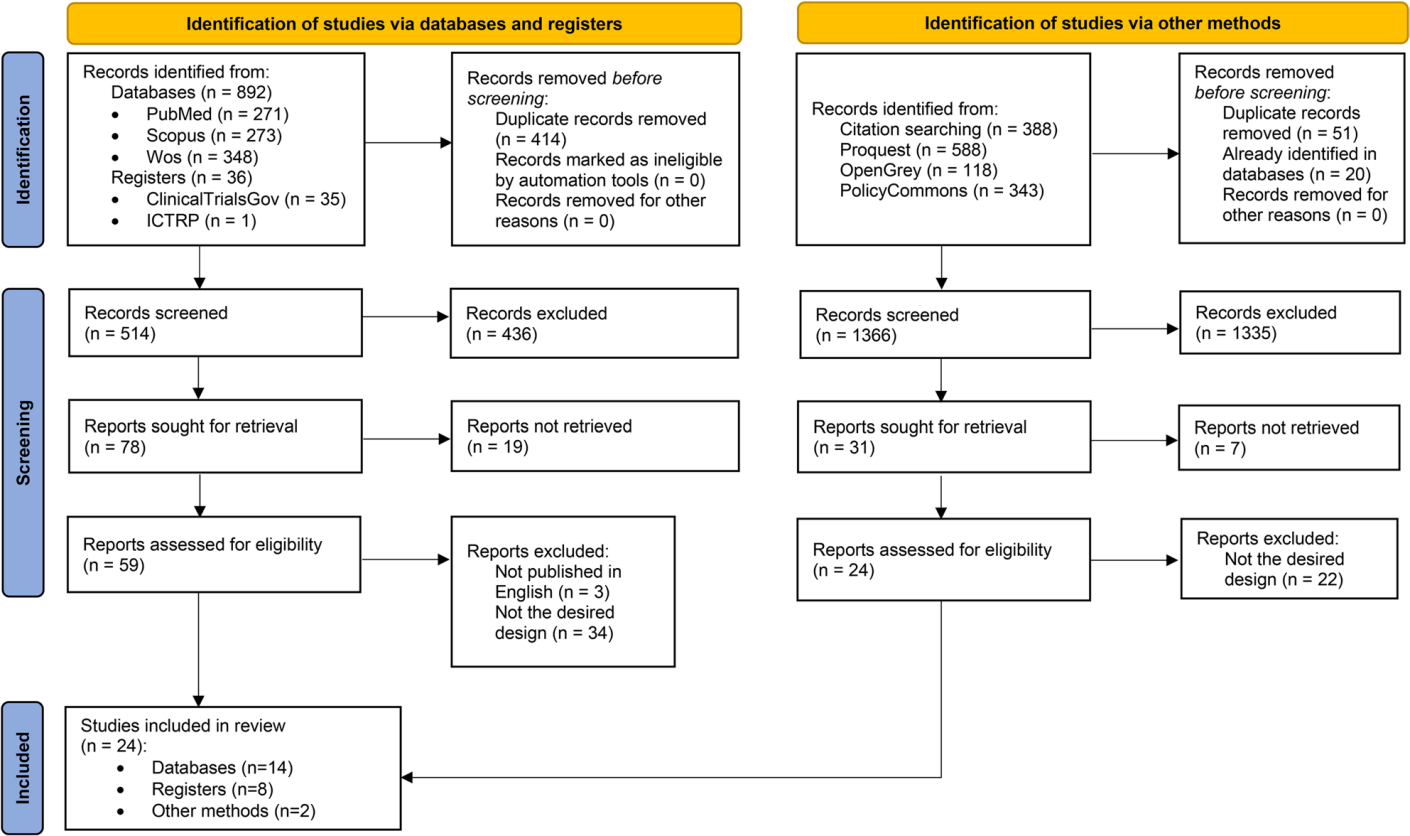
PRISMA 2020 flow diagram for systematic reviews which included searches of databases, registers and other sources

In this sense, studies were identified via other methods by searching the bibliography citation section of the 14 articles, identifying 388 references. In addition, 588 results were also identified from dissertations and theses, 118 grey literature documents and 343 policy reports. After analyzing the title and abstract, only 31 documents were included for retrieval, and after eliminating those that did not have the desired design, only 2 new studies were included as final results (Fig. 1).

### Data extraction process

Data extraction was performed independently by two reviewers (LLV and AMG), who analysed each study in detail to record the information. Disagreements on data extracted were resolved by involving a third reviewer to reach consensus (MBCM). Specifically, the following information was collected for each study (Supplementary file 2): study authors, year of publication, country, stroke type, sample size, patient age, time period, study design, type of cost analysis, indirect cost analysis approach, type of work productivity loss, perspective studied. In addition, estimated indirect cost values were compiled for each study according to type of stroke, age range of patients and type of work productivity loss. Information on trial registries can be found in Supplementary file 3.

### Risk of bias assessment

To assess the risk of bias (RoB) of the included studies, the Consolidated Health Economic Evaluation Reporting Standards (CHEERS) checklists were used [38]. In this regard, two reviewers (JFAL and VOV) assessed the studies by scoring each item with a 1 if it was met and a 0 if it was not met. Bar charts were then created with the relative frequencies of each of the responses given to each item (1 = Yes, 0 = No). Additionally, the compliance rate of each study was analysed and compared with that of the other studies in a boxplot using the statistical package SPSS Statistics v24.0 (IBM Corp., Armonk, NY).

## Results

### Characteristics of included studies

Following the search and selection process, a total of 16 articles and 8 trial registries were included in this systematic review. It should be noted that, once the selection process was completed, the 8 included trial registries were analysed in detail (Supplementary file 3), but all of them were excluded of data summarisation due to lack of available results. Thus, only the 16 articles identified through the electronic database search were considered for data summarisation, which is available in Table 1.

**Table 1:** Summary of study characteristics

Characteristics	Results	% (*n*)
Year of publication	Before 2020	56.3% (9)
	After 2020	43.7% (7)
Study location	Europe	43.7% (7)
	North America	43.7% (7)
	Asia	12.6% (2)
Stroke type	Ischemic	68.8% (11)
	Ischemic and hemorrhagic	25% (4)
	Ischemic, hemorrhagic and not specified	6.2% (1)
Sample size	Under 100	25% (4)
	100–500	18.6% (3)
	500–1000	25% (4)
	More than 1000	31.4% (5)
Patient age	Adults (> 18 years)	62.5% (10)
	18–64 years	25.1% (4)
	18–80 years	6.2% (1)
	40–61 years	6.2% (1)
Study design	Retrospective	62.5% (10)
	Prospective	37.5% (6)
Cost analysis type	Cost of illness	50% (8)
	Indirect cost analysis	50% (8)
Indirect cost analysis approach	Human capital	93.7% (15)
	Frictional	6.3% (1)
Work productivity loss	Absenteeism	43.6% (7)
	Absenteeism and informal care	25% (4)
	Absenteeism, presenteeism and informal care	18.8% (3)
	Absenteeism and presenteeism	6.3% (1)
	Informal care	6.3% (1)
Perspective	Societal	93.7% (15)
	Employer	6.3% (1)

Of the studies analyzed, 7 were performed in Europe, 7 in North America and 2 in Asia. Regarding the type of stroke, half of the studies (50%) analyzed patients with ischemic stroke, followed by 43.7% that included patients with both ischemic and hemorrhagic stroke, and a single study that included patients with unspecified stroke. Of these, 31.4% included more than 1,000 individuals, followed by 25% that included between 1,000 and 500, 18.6% that included between 500 and 100, and 25% that included less than 100. The age of the stroke patients included was over 18 years in all cases, with 62.5% of the studies considering all patients over 18 years, 25.1% of the studies considering patients between 18 and 64 years, a single study considering patients between 18 and 80 years, and another single study considering only patients between 40 and 61 years.

The study design was retrospective in 62.5% of the cases and prospective in 37.5% of the cases. Likewise, half of the studies performed a complete analysis of the cost of illness, including direct and indirect costs, while the other half only performed an analysis of indirect costs. Specifically, productivity loss was analyzed only from the point of view of absenteeism in 43.6% of the studies, followed by 25% of studies that analyzed it in terms of absenteeism and informal caregivers, 6.3% of studies that analyzed both absenteeism and presenteeism, and 6.3% that analyzed only informal caregivers. The vast majority of studies (93.7%) used the human capital approach for the analysis, and only one study used the frictional cost method. Also, most of studies approached the analysis from the societal perspective, while only one study considered the employer’s perspective.

### Description of included studies

Most of the included studies focused on analyzing the indirect costs of ischemic stroke from a societal perspective. This is the case of a retrospective study conducted in Sweden between 2006 and 2012 in which the indirect costs were estimated at €6,784 based on 47 days of absenteeism during the first year post stroke [16]. Similarly, a study conducted in Switzerland between 2015 and 2016 estimated that each case of ischemic stroke faced 97 lost work days equivalent to a loss in productivity valued at €7,589 during the first year post-stroke [39]. This study also estimated that informal caregivers of stroke patients lost a mean of 25 h per week caring for patients, resulting in a loss of work productivity valued at €10,635 in the first year post-stroke, and highlighting that these caregivers spent 12 h/week for patients with 3-month mRS = 0 − 2 and 59 h/week for patients with 3-month mRS = 3 − 5. This is consistent with the results of another retrospective study in China [40] which estimated that indirect costs during the first year after stroke were higher in patients with mRS = 3–5 at 3 months.

A prospective multicenter study estimated that the mean indirect cost during the first year after an ischemic stroke was €9,416 for absenteeism, €1,928 for presenteeism, and €2,428 for informal caregivers [41]. In this line of research, another study conducted in Portugal concluded that 73% of the productive time lost during the first year corresponds to hospitalization and patient leave, with 65 days of work lost amounting to €6,647.4 for absenteeism, €738.6 for presenteeism and €1,340 for informal caregivers [42]. In a study conducted in Romania in 2019 [43], indirect costs were estimated to account for 19.13% of total costs during the first year after stroke, although another study suggests that direct costs account for about half of the total costs in the first year after stroke [44].

Other studies compared indirect cost between the different types of stroke from a societal perspective. In this regard, a retrospective study in Denmark concluded that hemorrhagic strokes had higher indirect costs than ischemic and unspecified strokes during the first year, estimated at €10,720, €8,205 and €7,377 respectively [45]. Similarly, another study in Sweden concluded that hemorrhagic stroke patients had a mean of 145 lost working days compared to 95 days for ischemic stroke, and that patients with mRS = 5 at 3 months had costs up to eight times higher than patients with mRS = 0–2 [46]. Another study in Turkey also found that indirect costs due to absenteeism were higher in hemorrhagic stroke [47].

A retrospective study conducted in the United States between 2003 and 2014 estimated a total value of $38.1 billion for indirect costs, with absenteeism during the first year post-stroke representing a productivity loss of $9,393 per patient [48]. Another US study also confirmed that indirect costs were higher in hemorrhagic strokes than in ischemic strokes [49]. This study also highlighted that hemorrhagic stroke patients aged 18–44 years had a higher indirect cost of $14,945 compared to $10,307 for ischemic strokes. Similar results were obtained in a retrospective study in Canada where it was found that lost productivity during the first year after stroke in patients aged 40 to 61 years was higher in hemorrhagic strokes than in ischemic strokes [50]. It was also concluded that indirect costs were higher for patients who had more comorbidities, a longer hospital stay, and required mechanical ventilation during their hospital stay. Between 2016 and 2017, a study also conducted in Canada estimated a mean of $9,048 for absenteeism and $1,250 for presenteeism [51], with the most severe stroke patients (mRS > 1 at 3 months) losing a mean of 83 working days, compared to 45 days lost by patients with mRS ≤ 1 at 3 months.

Considering the informal caregiver perspective, a 2018 prospective US study [52] found that patients with post-stroke spasticity caused their informal caregivers a loss of work productivity of $10,020 during the first year after the stroke onset. Specifically, informal caregivers spent a mean of 19, 38 and 49 h per week caring for patients with mild, moderate, and severe disability, respectively. On the other hand, another US study that retrospectively analyzed indirect cost loss from the employer perspective estimated that monthly hours lost due to work absenteeism in patients with cardiovascular events and related clinical procedures reached 23.1, resulting in a productivity loss of $8,376 per patient during the first year of follow-up [53]. In addition, ischemic stroke was the third most common cause of productivity loss behind myocardial infarction and heart failure.

### Synthesis of results

A comparative analysis of the results obtained from the 15 studies that examined indirect costs during the first year after stroke from a societal perspective was conducted. The estimated indirect cost from each study were compiled based on the type of stroke and the type of work productivity loss, all expressed in US dollars (USD) according to the exchange rate for the year of each study. These values ​​were updated to 2025 at an annual rate of 5%. Analysis was carried out with SPSS Statistics v24.0 (IBM Corp., Armonk, NY).

**Fig. 2:**
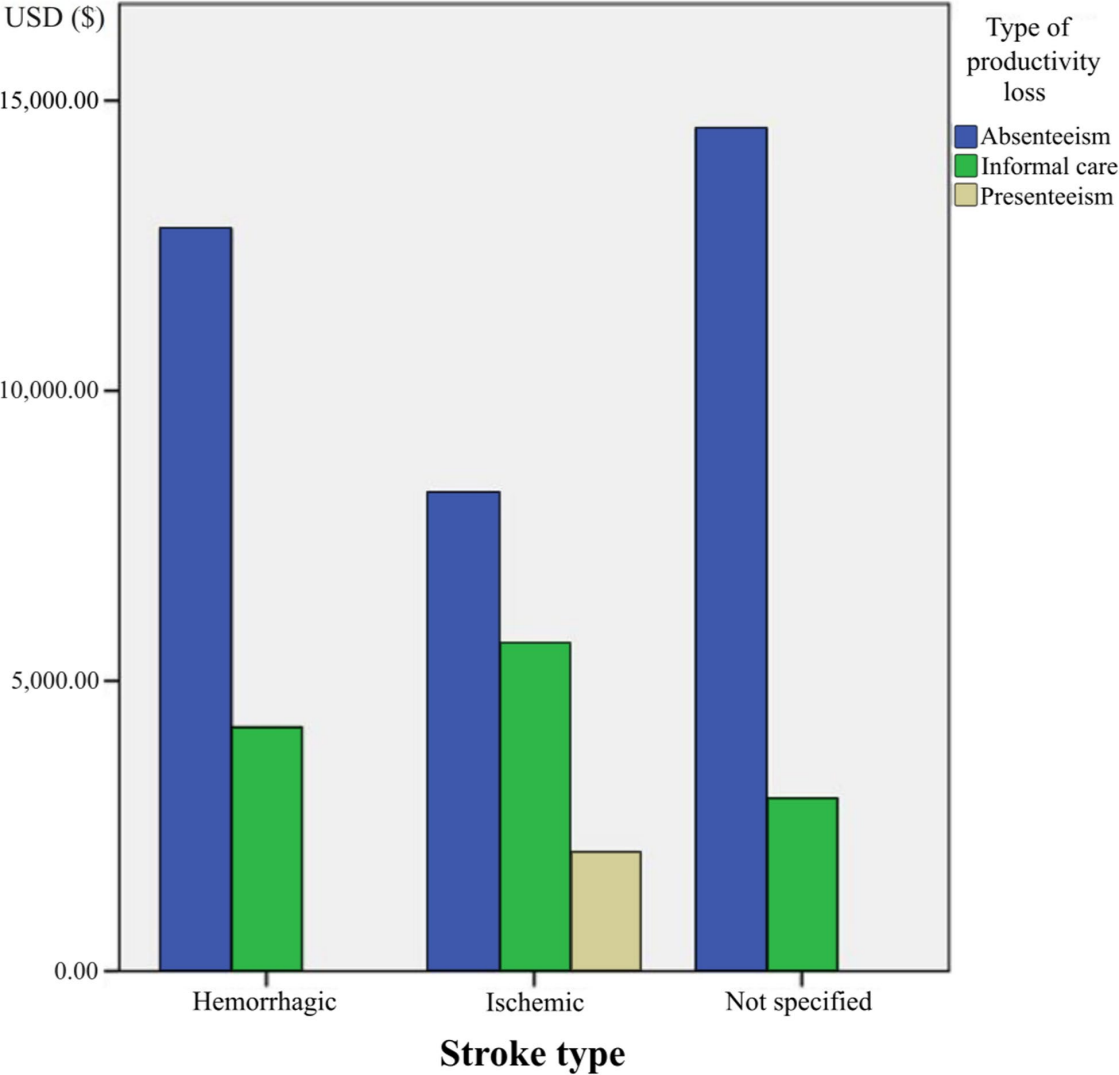
Productivity loss in USD according to the type of stroke

The overall results indicated that work productivity loss in all adult’s patients had a mean value of $6057.98 (SD: 3043.06) during the first post-stroke year, with these indirect costs representing a mean of 35.1% of the total cost according to studies that performed a cost-of-illness analysis. Regarding the age of the patients, those between 18 and 44 years experienced the greatest loss of productivity, with a mean of $16,114.33 (SD: $4,185.64). Patients aged 65–84 years had a mean loss of productivity of $7,380.09 (SD: $234.33) and from the age of 85 years was valued to $3,918.82 (SD: $899.76), mainly due to the loss of productivity of the informal caregiver. On the other hand, the results of the cross-tabulation analysis according to the type of stroke are presented in Fig. 2.

The mean value of productivity loss during the first year after stroke due to absenteeism was higher in hemorrhagic strokes ($12,783.17, SD: $7,041.05) than in ischemic strokes ($8,239.34, SD: $5,223.18). The mean value of productivity loss during the first year after stroke due to presenteeism was $2,039.36 (SD: $1,134.17) in ischemic strokes, but could not be compared with that for hemorrhagic strokes due to lack of available data. On the other hand, unspecified strokes, compared to other stroke types, had a higher mean value of lost productivity due to absenteeism, reaching up to $14,514.68, although these data only come from one study [45]. Lastly, the mean value of lost productivity during the first year after stroke for informal caregivers was higher for ischemic strokes ($5,628.26, SD: $4,682.27) than for hemorrhagic strokes ($4,205.25, SD: $2,998.03).

Based on the overall analysis of lost work days during the first year after stroke onset, ischemic stroke patients lost a mean of 62.1 days due to absenteeism (SD: 20.1) and 10.1 days due to presenteeism (SD: 6.1), while their informal caregivers lost a mean of 19.1 days (SD: 8.2). Regarding hemorrhagic strokes, these caused up to 145 workdays lost in total, which is higher than the loss caused by ischemic strokes.

**Fig. 3:**
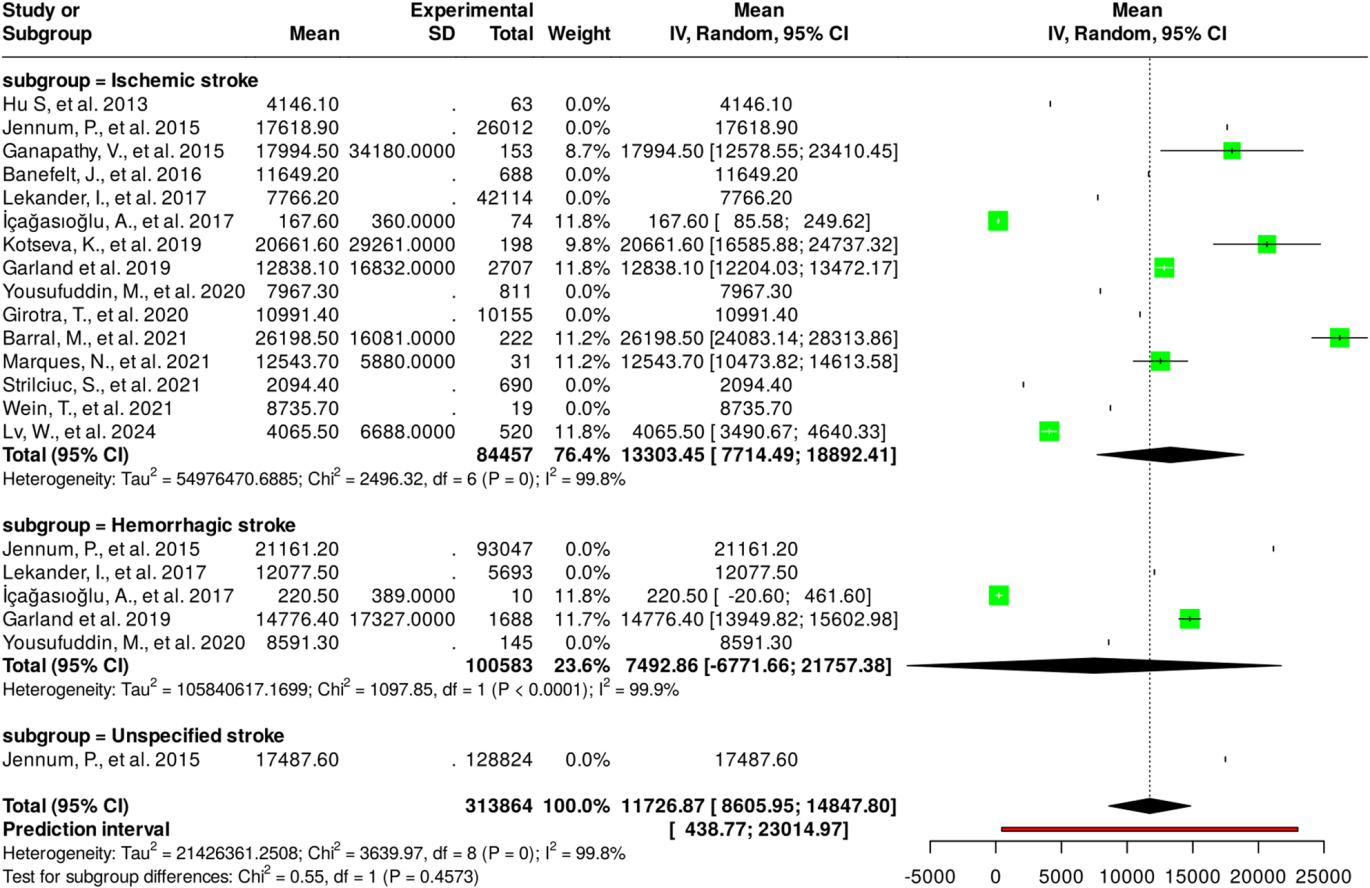
Results of the heterogeneity analysis

Considering the mean value of the total indirect cost of patients and their informal caregivers in each study, an analysis was performed with heterogeneity tests [29]. This analysis was performed with the 15 studies included in the comparative analysis, but only 7 studies presented standard deviation (SD) values for the mean values. All studies were classified into subgroups according to the type of stroke analyzed: ischemic, hemorrhagic, or unspecified stroke, taking into account that those studies that analyzed several types of stroke were placed in the subgroups corresponding to each type. The results of the heterogeneity analysis are presented in Fig. 3.

The results indicate that there is overall heterogeneity and also heterogeneity within each subgroup. According to the analysis performed using the random effects model with the inverse variance method, the raw mean summary (RMS) was 11,726.87 with a 95% confidence interval of 8,605.95 to 14,847.8. In this sense, significant heterogeneity was detected (*p* < 0.05), suggesting inconsistent effects in magnitude and/or direction. In addition, the I₂ value indicates that 99.8% of the variability between studies is due to heterogeneity and not to chance (Tau2 = 21426361.2508; Chi2 = 3639.97, df = 8 (*P* = 0); I2 = 99.8%). Regarding the heterogeneity of the subgroups, the ischemic stroke and hemorrhagic stroke subgroups show significant heterogeneity (*p* < 0.05), while the unspecified stroke subgroup consists of only one study. As for the test for subgroup differences, no significant differences were observed (*p* > 0.05), suggesting that the variability of values between subgroups is small (Chi2 = 0.55, df = 1, *P* = 0.4573).

### Risk of bias

**Fig. 4:**
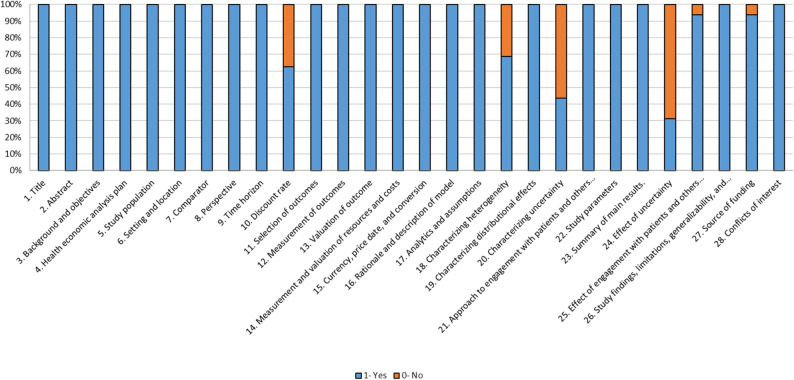
Compliance of included studies with CHEERS

The risk of bias of the included studies was assessed according to their compliance with each of the CHEERS items, assigning a score of 1 when the study met the condition and 0 when it did not. The risk of bias assessment of the included studies is shown in Fig. 4. Studies mostly fulfilled all the items considered, except those relating to the discount rate, characterisation of heterogeneity and uncertainty, effect of uncertainty, engagement with patients and others affected by the study, and source of funding.

**Fig. 5:**
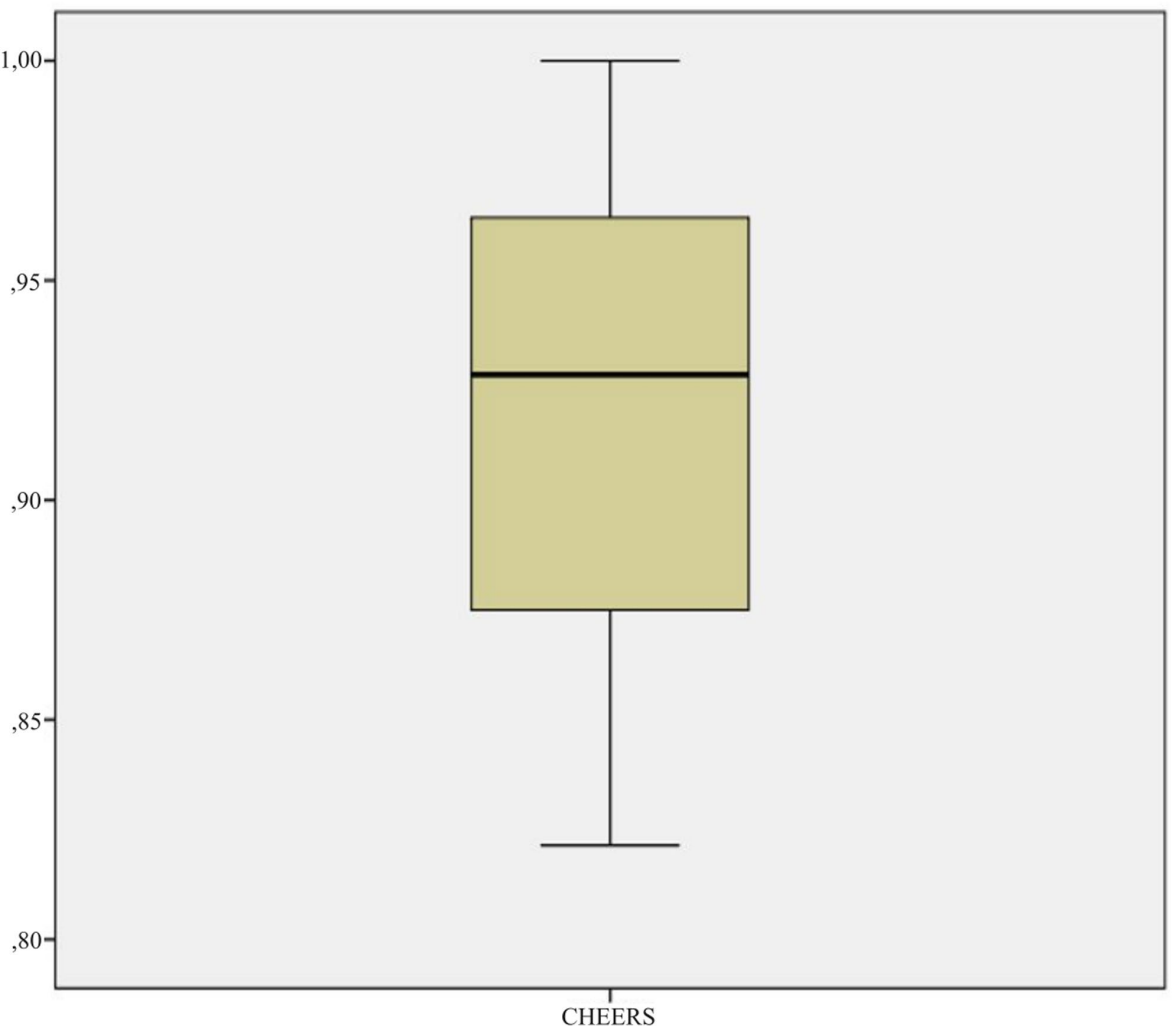
Boxplot analysis of assigned scores to CHEERS

Subsequently, a statistical analysis was performed to assess the compliance rate compared between all studies (Fig. 5). Specifically, the compliance rate for each study was calculated as the mean of the score given to the CHEERS items (Yes = 1 or No = 0). The median compliance rate across studies was 0.929. In this regard, 75% of the included studies had a compliance rate higher than 0.866, with some studies having a compliance rate of 1.00 [39, 42, 47]. On the other hand, four studies [43, 44, 51, 52] had a compliance rate below 0.8661 and one study had the lowest compliance rate at 0.820 [44].

## Discussion

Although strokes are more common in older people, the number of cases in working-age adults has increased and will continue to increase in recent years [13]. This will result in a significant number of stroke survivors with physical and/or cognitive disabilities, which implies a large indirect cost relative to work productivity loss. In addition to working-age patients, these indirect costs also arise for patients of all ages due to the impact on work productivity of their informal caregivers. However, indirect costs are often left out of socioeconomic impact analyses [26] and existing reviews on this topic are outdated [27]. Therefore, we conducted an updated systematic review focusing on indirect costs related to loss of work productivity during the first year after stroke to highlight their importance and encourage their inclusion in future socioeconomic impact studies.

A total of 8 trial registries and 16 studies published between 2013 and 2024 and conducted in Europe, North America and Asia were identified. These studies mainly analyzed ischemic stroke. A larger number of retrospective studies have been conducted, most with a sample of > 500 individuals. To analyse indirect costs, the vast majority of studies used the human capital approach. Productivity loss was analysed only in terms of absenteeism in 43.6% of the studies, while 25% of the included studies analysed it in terms of both absenteeism and informal caregiver. Only 3 studies included also presenteeism.

Included studies mostly analyzed the indirect costs related to work productivity loss due to absenteeism, estimating a higher mean value in hemorrhagic strokes ($12,783.17) than in ischemic strokes ($8,239.34). This could be due to the fact that hemorrhagic strokes implies a worse functional prognosis of patients after discharge, more intensely affecting their ability to perform their previous jobs. Studies analyzing ischemic strokes estimated a mean of 62.1 workdays lost due to absenteeism and 10.1 days due to presenteeism. Patients aged 18–44 years experienced the highest productivity loss during the first year after stroke, with a mean value of $16,114.33. Patients with worse functional status (higher mRS) at 3 months experienced a higher work productivity loss, as well as a greater need for informal care [42]. Similarly, productivity loss was higher in patients with atrial fibrillation [44], spasticity and hyperlipidemia prior to stroke [43]. In contrast, patients with more convenient access to urgent medical care and follow-up after stroke in urban and rural areas had lower indirect costs [40]. Productivity loss by informal caregivers was higher in ischemic strokes ($5,628.26) than in hemorrhagic strokes ($4,205.25). These losses were greater when there was no nursing home nearby [43] and lower where more than one informal caregiver was available, due to shared tasks [52].

To assess the risk of bias of the articles included in the review, standardised criteria based on CHEERS were followed, highlighting that most of the studies had a rate of compliance close to 90%. However, a limitation is the heterogeneity of the studies in terms of the age of the patients analyzed and the lack of recall in answering the surveys by patients or their informal caregivers [16]. There was also some difficulty in verifying whether the costs of informal care of stroke patients were not due to stroke but to other health conditions, co-morbidities or simply as a consequence of advanced age [45]. Furthermore, the term indirect cost was considered in its broadest definition during the article search process, which may limit the relevance of analyzing each component of work productivity lost separately.

The results of this review are in line with those of another review published in 2014 [27]. In this regard, the most widely used indirect cost analysis approach continues to be the human capital approach from a social perspective. In this sense, the indirect cost depends on study design and duration of follow-up, estimating in 2014 a mean loss of work productivity of between $2,960 and $22,243 per stroke patient per year, values in line with the results obtained. In addition, patient presenteeism and informal care were the most omitted components in estimating the indirect costs of stroke [39].

Nevertheless, while the 2014 review [27] used a less detailed study selection methodology, our review followed the PRISMA methodology [28] with a comprehensive explanatory diagram and used a broader database source and including registries and the identification of studies via other methods. Furthermore, the 2014 review [27] defined the inclusion of indirect cost types based on lost work productivity due to mortality, morbidity, or informal caregivers, whereas our study considered indirect costs due to absenteeism, presenteeism, or informal caregivers, allowing us to include the importance of presenteeism following illness. On the other hand, the time horizon of the design of the studies included was broader in the 2014 review [27], as it included studies that assessed indirect costs from the first months after stroke to those that considered lifetime costs. However, in our review, we included studies that assess indirect costs during the first year after stroke in order to perform a comparative analysis of all the results.

Thus, our study conducted an aggregate analysis of the data to compare indirect costs according to the type of stroke analyzed, the type of work productivity loss, and patient age groups, while the 2014 review [27] only presented the data from each study in a descriptive manner. Furthermore, with regard to the assessment of heterogeneity, the 2014 review [27] briefly discussed the wide variety of study characteristics, while our systematic review, in addition to analyzing the characteristics of the studies, also carried out a detailed statistical analysis of heterogeneity on the mean values of total indirect costs, observing a significant variability between the results obtained among all the studies included. In addition, our systematic review performed a risk of bias analysis of the included studies using the CHEERS methodology, while the 2014 review did not perform this type of analysis in this regard.

It is also worth noting that the policy reports identified highlighted the importance of the indirect costs of stroke on the socioeconomic impact of this disease. In 2024, a report by the American Heart Association (AHA) [54] stated that between 2017 and 2020, the total costs of cardiovascular disease in USA were estimated at $422.3 billion, of which $168.0 billion corresponded to lost productivity. Another report in USA [55] estimated that annual productivity losses due to cardiovascular disease will increase by 54% by 2050. Another reports in USA [56, 57] highlights that the costs of informal caregiving constitute more than half of the indirect costs. On the other hand, a 2020 report by the Stroke Alliance for Europe (SAFE) [58] predicted that by 2040, the number of people living with stroke will increase by 35% reaching 12 million individuals. According to Action Plan for Stroke in Europe 2018–2030 [59] one in four strokes occur in people of working age under 65 and defines the importance of early supported discharge to provide post-stroke patients specialized rehabilitation so that they can return to work. In China, a 2019 report [60] highlights that the highest indirect cost among patients aged 50 to 84.

Therefore, when analysing the socioeconomic impact of stroke, it is essential to include the indirect costs in terms of lost productivity of the patient due to absenteeism and presenteeism, as well as those of the informal caregiver [41]. Future research should obtain precise data on the risk factors associated with loss of productivity after stroke. It is also interesting to further investigate the difference in indirect costs as a function of treatment variables, and the need for informal caregivers in patients before and after stroke. Furthermore, the magnitude of the lost work productivity associated with stroke depends on the country’s level of economic and social development. In developed countries, the stroke population is older and informal caregiving is often supported by social protection systems that offer pensions, sickness benefits, and other types of assistance. In contrast, in developing countries, stroke occurs more frequently in younger people and families primarily assume aftercare with limited financial support from social assistance systems. For all of this, it is essential to promote studies of socioeconomic impact of stroke in different contexts, to optimize the allocation of health resources.

## Conclusions

The studies identified in this systematic review reinforce the idea that indirect costs represent a significant part of the economic burden of stroke. Moreover, the effect on productivity losses varies according to their typology, with hemorrhagic strokes representing a higher indirect cost than ischemic strokes. In this regard, hemorrhagic strokes cause a worse functional prognosis after discharge, which affects patients’ ability to perform their previous work activity. Notably, patients aged 18 to 44 years experienced the greatest loss of productivity during the first year after stroke, which represents the highest indirect cost compared to other age groups. Meanwhile, informal caregivers of post-stroke patients experience their highest loss of productivity in cases of ischemic stroke, since these need more care during the first year of recovery. Although there is a high heterogeneity among existing studies, in terms of characteristics and results.

Nevertheless, indirect costs remain underestimated in socioeconomic impact analyses, highlighting the need for health systems to promote efforts to understand and reduce the loss of productivity for both patients and informal caregivers due to stroke, through improvements along all the continuum of stroke care: prevention, acute phase and post-stroke care, to achieve better functional status and quality of life for patients. Therefore, a holistic approach to analysing the indirect costs of stroke is necessary to increase understanding and awareness and improve the efficiency of the healthcare system. 

## Supplementary Information

Supplementary Material 1. Supplementary File 1. Search strategy used for each electronic database.

Supplementary Material 2. Supplementary File 2. Information collected for each study.

Supplementary Material 3. Supplementary File 3. Characteristics of trial registers.

## Data Availability

No datasets were generated or analysed during the current study.

## Abbreviations

CHEERS: Consolidated Health Economic Evaluation Reporting Standards.
ICTRP: International Clinical Trials Registry Platform.
MeSH: Medicine’s Medical Subject Headings.
PRISMA: Preferred Reporting Items for Systematic Reviews and Meta-Analyses.
RoB: Risk of bias.
Wos: Web of Science.
WHO: World Health Organization.
