# Supplementary material for: A systematic review on indirect costs related to loss of productivity after stroke
Source: Health Econ Rev. 2026 Jan 31;16:25. doi: 10.1186/s13561-026-00727-x (PMC12947508; doi:10.1186/s13561-026-00727-x)
Supplement: Supplementary file 1 — Supplementary Material 1. Supplementary File 1. Search strategy used for each electronic database. [file 13561_2026_727_MOESM1_ESM.docx]

**Supplementary File 1**

**Search strategy used for each electronic database**

| **Database** | **Search strategy** |
| --- | --- |
| PubMed | ("Stroke" OR "Brain Infarction") AND ("Indirect Costs" OR "Indirect Costs Analysis" OR "Indirect Costs Measurement" OR "Indirect Costs Allocation") |
| Scopus | TITLE-ABS-KEY(("Stroke" OR "Brain Infarction") AND ("Indirect Costs" OR "Indirect Costs Analysis" OR "Indirect Costs Measurement" OR "Indirect Costs Allocation")) |
| WoS | TS=(("Stroke" OR "Brain Infarction") AND ("Indirect Costs" OR "Indirect Costs Analysis" OR "Indirect Costs Measurement" OR "Indirect Costs Allocation")) |

TITLE-ABS-KEY = Title-Abstract-Keywords; WoS = Web of Science; TS = Topic
