# Supplementary material for: A systematic review on indirect costs related to loss of productivity after stroke
Source: Health Econ Rev. 2026 Jan 31;16:25. doi: 10.1186/s13561-026-00727-x (PMC12947508; doi:10.1186/s13561-026-00727-x)
Supplement: Supplementary file 2 — Supplementary Material 2. Supplementary File 2. Information collected for each study. [file 13561_2026_727_MOESM2_ESM.docx]

**Supplementary file 2**

**Information collected for each study**

| **Study authors** | **Year of publication** | **Country** | **Stroke type** | **Sample size** | **Patient age** | **Period** | **Study design** | **Cost analysis type** | **Indirect cost analysis approach** | **Type of work productivity loss** | **Perspective** |
| --- | --- | --- | --- | --- | --- | --- | --- | --- | --- | --- | --- |
| Hu S, et al. (2013) | 2013 | China | Ischemic | 63 | Adults (>18 years) | 2010-2011 | Retrospective | Cost of illness | Human capital | Absenteeism and informal care | Societal |
| Jennum, P., et al. (2015). | 2015 | Denmark | Ischemic, hemorrhagic and not specified | Ischemic: 93047  Hemorrhagic: 26012  Not specified: 128824 | Adults (>18 years) | 1997-2009 | Retrospective | Cost of illness | Human capital | Absenteeism and informal care | Societal |
| Song et al. (2015) | 2015 | EE.UU. | Ischemic | 5808 | 18–64 years | 2002-2011 | Retrospective | Indirect cost analysis | Frictional | Absenteeism | Employer |
| Ganapathy, V., et al. (2015). | 2015 | EE.UU. | Ischemic and hemorrhagic | 153 | Adults (>18 years) | 2008 | Prospective | Indirect cost analysis | Human capital | Informal care | Societal |
| Banefelt, J., et al. (2016). | 2016 | Sweden | Ischemic | 688 | 18-64 years | 2006- 2012 | Retrospective | Indirect cost analysis | Human capital | Absenteeism | Societal |
| Lekander, I., et al. (2017). | 2017 | Sweden | Ischemic and hemorrhagic | Ischemic: 42114  Hemorrhagic: 5693 | 18-64 years | 2007-2010 | Retrospective | Cost of illness | Human capital | Absenteeism | Societal |
| İçağasıoğlu, A., et al. (2017). | 2017 | EE.UU. | Ischemic and hemorrhagic | Ischemic: 74  Hemorrhagic: 10 | Adults (>18 years) | 2014 | Prospective | Cost of illness | Human capital | Absenteeism | Societal |
| Kotseva, K., et al. (2019). | 2019 | Seven European countries | Ischemic | 198 | Adults (>18 years) | 2016-2017 | Prospective | Indirect cost analysis | Human capital | Absenteeism, presenteeism and informal care | Societal |
| Garland et al. (2019) | 2019 | Canada | Ischemic | 4395 | 40–61 years | 2008-2010 | Retrospective | Indirect cost analysis | Human capital | Absenteeism | Societal |
| Yousufuddin, M., et al. (2020). | 2020 | EE.UU. | Ischemic and hemorrhagic | Ischemic: 811  hemorrhagic: 145 | 18–44 years  45–64 years  65–84 years  ≥85 years | 2003-2014 | Retrospective | Cost of illness | Human capital | Absenteeism and informal care | Societal |
| Girotra, T., et al. (2020) | 2020 | EE.UU. | Ischemic | 10155 | 18-24 years  25-44 years  45-64 years | 2003-2014 | Retrospective | Cost of illness | Human capital | Absenteeism | Societal |
| Barral, M., et al. (2021). | 2021 | Swiss | Ischemic | 222 | Adults (>18 years) | 2015-2016 | Prospective | Indirect cost analysis | Human capital | Absenteeism, presenteeism and informal care | Societal |
| Marques, N., et al. (2021). | 2021 | Portugal | Ischemic | 31 | Adults (>18 years | 2016-2017 | Prospective | Indirect cost analysis | Human capital | Absenteeism, presenteeism and informal care | Societal |
| Strilciuc, S., et al. (2021). | 2021 | Romania | Ischemic | 690 | Adults (>18 years) | 2019 | Retrospective | Cost of illness | Human capital | Absenteeism and informal care | Societal |
| Wein, T., et al. (2021). | 2021 | Canada | Ischemic and hemorrhagic | Ischemic: 19 Hemorrhagic: 1 | Adults (>18 years) | 2016-2017 | Prospective | Indirect cost analysis | Human capital | Absenteeism and presenteeism | Societal |
| Lv, W., et al. (2024) | 2024 | China | Ischemic | 520 | 18-80 years | 2017-2018 | Retrospective | Cost of illness | Human capital | Absenteeism | Societal |
