# Supplementary material for: A systematic review on indirect costs related to loss of productivity after stroke
Source: Health Econ Rev. 2026 Jan 31;16:25. doi: 10.1186/s13561-026-00727-x (PMC12947508; doi:10.1186/s13561-026-00727-x)
Supplement: Supplementary file 3 — Supplementary Material 3. Supplementary File 3. Characteristics of trial registers. [file 13561_2026_727_MOESM3_ESM.docx]

**Supplementary file 3**

**Characteristics of** **trial registers**

| **ID Number** | **Title** | **Start Date** | **Country** | **Economic analysis** | **Status** |
| --- | --- | --- | --- | --- | --- |
| NCT02212145 | Follow-up of Sollentuna Prevention Program (SoPP) | 2014-08-08 | Sweden | Health economic aspects for impact on indirect costs | Unknown status, no  results  available |
| NCT02683213 | Efficacy of Fluoxetine - a Trial in Stroke (EFFECTS) | 2016-02-02 | Sweden | Direct and indirect costs will be estimated at 3-month, 6- month and 1 year. | Completed but no  results  available |
| NCT03605355 | Feasibility Study on the Medical and Economic Consequences of Outpatient Management of TIAs and Minor Strokes | 2018-07-19 | France | Direct medical and non-medical costs and indirect costs | Unknown status, no  results  available |
| NCT03930121 | Transcranial Direct Current Stimulation to Enhance Training Effectiveness in Chronic Post-Stroke Aphasia | 2019-04-29 | Germany | Direct and indirect costs during the 12-month study period. | Recruiting, no  results  available |
| NCT04457908 | The Effectiveness and Cost Effectiveness of Intelligent Assessment of Gait Disorder in Silent Cerebrovascular Disease (ACCURATE-1) | 2020-07-07 | China | Direct and indirect costs of the intelligent system and clinicians. | Unknown status, no  results  available |
| NCT05970367 | Intensive Motor Rehabilitation With Technology for Patients With Central Neurological Disease (INTeRAcT) | 2023-08-01 | Belgium | Direct and indirect costs. | Recruiting, no  results  available |
| NCT06547827 | Rehabilitation with and Without Robot and Allied Digital Technologies in Stroke Patients (StrokeFit4) | 2024-07-29 | Italy | Evaluate the costs (direct healthcare costs, direct non-healthcare costs, indirect costs ). | Recruiting, no  results  available |
| NCT06443840 | Impact of a Self-rehabilitation and Tele-rehabilitation Program on the Post-stroke Care Pathway (AUTONHOME) | 2024-06-05 | France | Study of direct and indirect costs | Recruiting, no  results  available |
